# Supplementary material for: Human and mouse essentiality screens as a resource for disease gene discovery
Source: Nat Commun. 2020 Jan 31;11:655. doi: 10.1038/s41467-020-14284-2 (PMC6994715; doi:10.1038/s41467-020-14284-2)
Supplement: Supplementary file 1 — Supplementary Information [file 41467_2020_14284_MOESM1_ESM.pdf]

Supplementary Information

Human and mouse essentiality screens as a resource for disease gene discovery

Cacheiro, Muñoz-Fuentes et al.

Content

Supplementary Figures 1-9

Supplementary Tables 1-9

Supplementary References

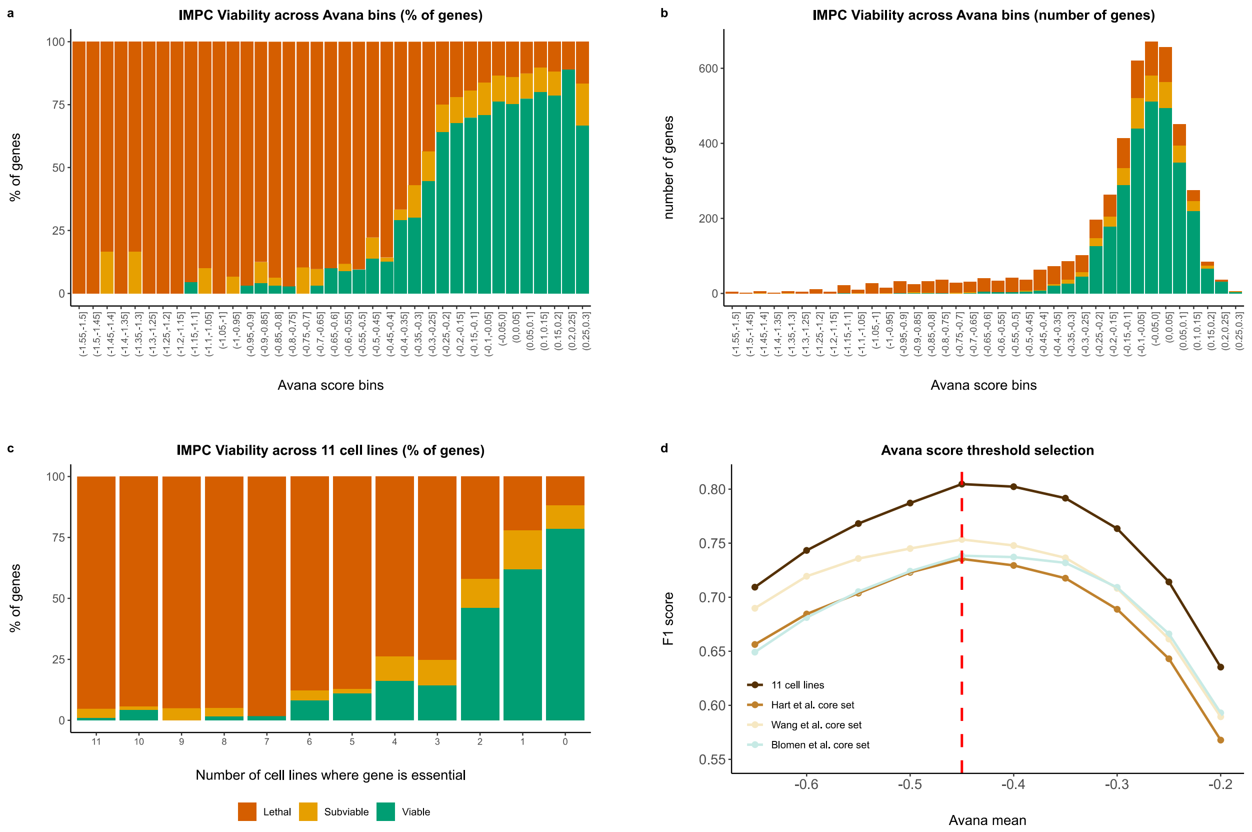

Supplementary Figure 1. Selection of mean Avana score threshold to identify essential genes.

a) and b) Distribution of IMPC viability categories across bins of mean Avana scores. Bar plots showing the percentage (a) and numbers (b) of lethal, subviable and viable mouse-to-human orthologous genes across mean Avana score bins comprising 4,446 genes for which there was IMPC viability data, a good confidence orthologue and an Avana viability score (release 18Q3 of August 2018 for 17,634 genes in 485 cell lines). For genes with an Avana mean score ≤ -0.45, the mouse null homozygotes were lethal in almost all cases, while genes with an Avana mean score > -0.45 presented lethal, subviable or viable phenotypes. **c) IMPC Viability categories across 11 cell lines.** A similar pattern was observed when a different source consisting on 11 cell lines from 3 different studies was used (Munoz-Fuentes, et al. [1]). **d) F1 scores for the comparison with previous datasets.** F1 scores derived from the confusion matrices considering different Avana mean scores and the classification in essential versus non-essential genes from previous studies. An Avana score cut-off of -0.45, which maximises the F1 scores across the different datasets, was selected, so that all genes with an Avana mean score below or equal to -0.45 were considered essential.

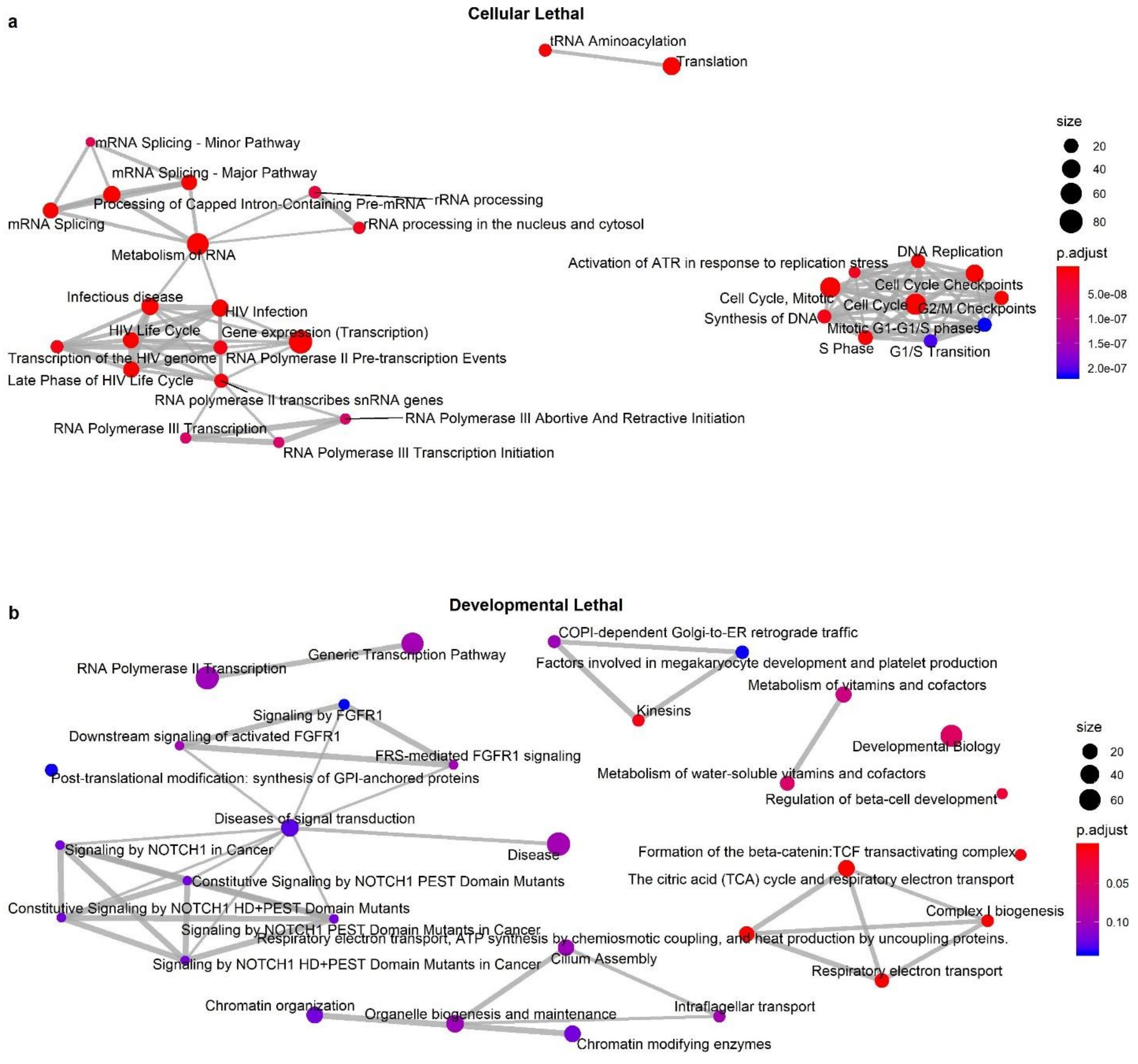

Supplementary Figure 2. Reactome pathways enrichment analysis.

Reactome pathways enrichment results for the set of CL a) and DL genes (b). Enriched Reactome pathways[2] identified using the set of IMPC mouse-to-human orthologues with FUSIL categorisation as a reference (Table 1). Significant results after correcting for multiple testing (BH) for all FUSIL categories are shown in Supplementary Data 2.

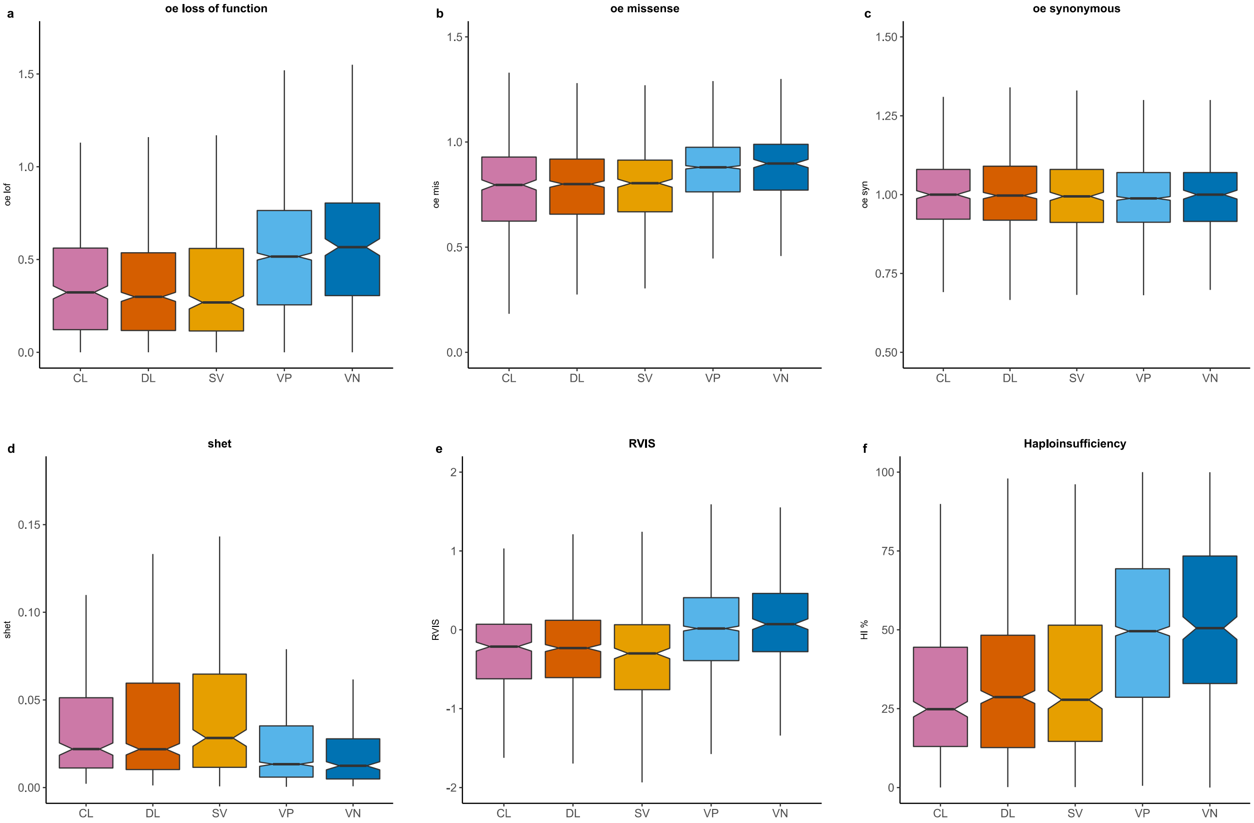

Supplementary Figure 3. Distribution of different constraint scores derived from human population sequencing data across the five FUSIL categories established in this study. a), b) and c) Observed versus expected (o/e) ratio of gnomAD 2.1 scores[3,4].

a) Distribution of o/e LoF scores; lower scores indicate more intolerance to LoF. **b)** Distribution of o/e missense scores; lower scores indicate more intolerance to missense variation. **c)** Distribution of o/e synonymous scores; lower scores indicate more intolerance to synonymous variation. **d) Estimates of selection against heterozygous loss of gene function.** The selective effects for heterozygous protein-truncating variants (shet) were obtained from the supplementary material of Cassa, et al. [5], with higher values indicating more intolerant to variation**. e) Residual Variance Intolerance Score.** Distribution of the Residual Variation Intolerance Score (RVIS; version CCDSr20)[6], with lower values indicating more intolerance. **f) Haploinsufficiency percentage score.** Haploinsufficiency score as a percentage (HI%), computed by the Deciphering Developmental Disorders (DDD) consortium[7]. High ranks (e.g. 0-10%) indicate a gene is more likely to exhibit haploinsufficiency, low ranks (e.g. 90-100%) indicate a gene is more likely to not exhibit haploinsufficiency. For figures a), b), c), d), e) and f): center line, median; notch, CI around the median; box edges, interquartile

range, 75th and 25th percentile respectively; whiskers, 1.5 times the interquartile range; outliers not shown. Significance of pairwise comparisons for all the constraint metrics are shown in Supplementary Table 5. CL, cellular lethal, pink; DL, developmental lethal, orange; SV, subviable, yellow; VP, viable with phenotypic abnormalities, light blue; VN, viable with normal phenotype, dark blue**.**

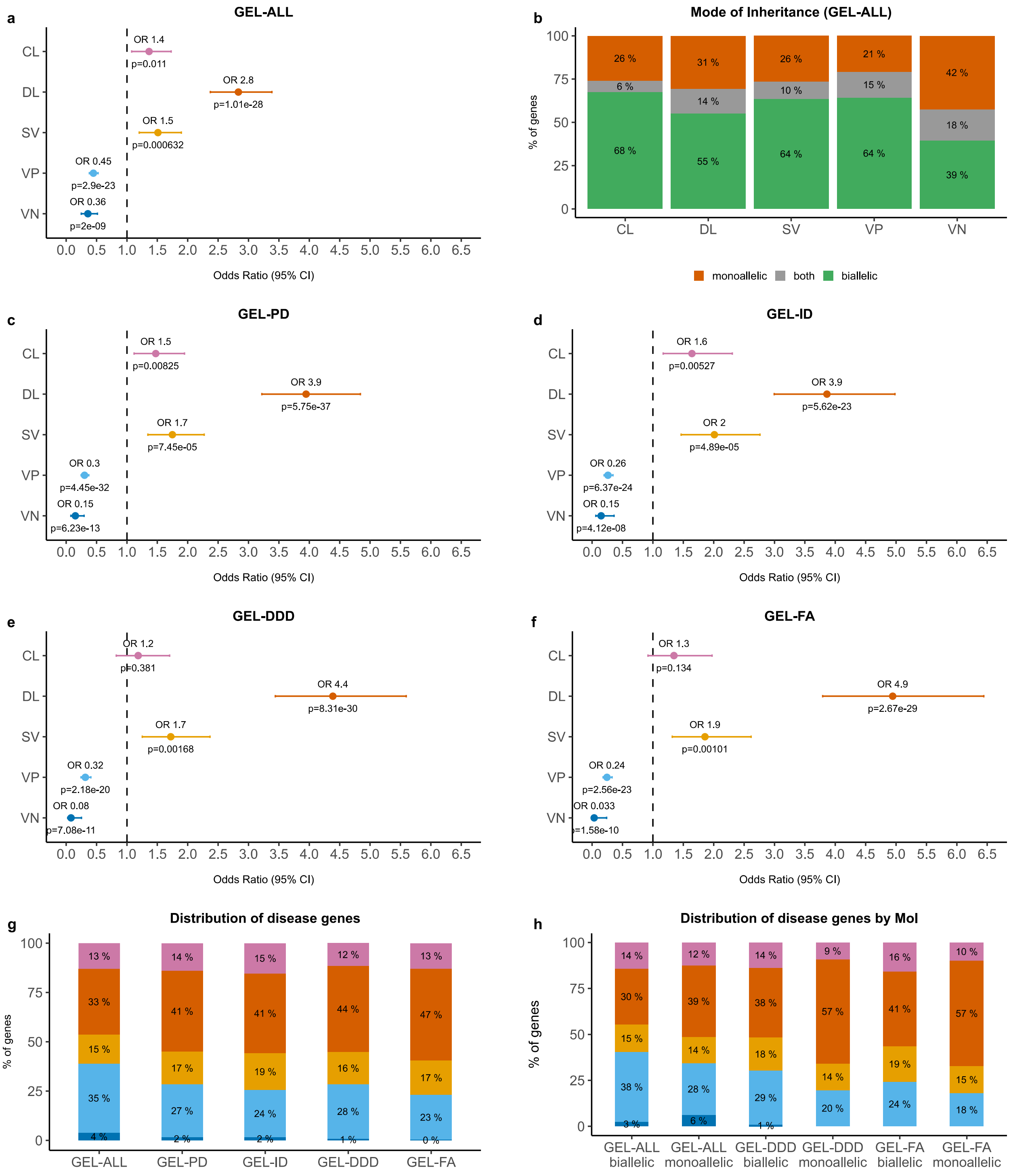

Supplementary Figure 4. Human diagnostic-grade genes and FUSIL bins.

a) Enrichment analysis of diagnostic-grade genes. Set of green genes included in any Genomics England gene panel (PanelApp). **b) Distribution of diagnostic-grade genes according to mode of inheritance**. Green genes with the associated mode of inheritance

according to PanelApp (only “monoallelic”, “biallelic” or “both” categories were considered). **c) Enrichment analysis of genes associated to paediatric disorders.** Set of “green” genes from GEL Paediatric disorders gene panel. **d) Enrichment analysis of genes associated to intellectual disability.** Set of green genes from GEL Intellectual disability gene panel. **e) Enrichment analysis of genes associated to developmental disorders.** Set of green genes from GEL DDG2P panel, which a contains a subset of DDG2P genes with one of the following levels of evidence: Confirmed or both DD and IF. **f) Enrichment analysis of genes associated to fetal anomalies.** Set of green genes from GEL fetal anomalies panel, which contains a subset of genes associated to developmental disorders developed by PAGE (Prenatal Assessment of Genomes and Exomes) with a confirmed disease confidence rating that underwent additional review and curation. **g) Distribution of disease (diagnostic grade) genes**. Bar plots show the percent distribution of different sets of green genes from PanelApp among the different FUSIL categories. **h) Percent distribution of disease genes by mode of inheritance**. Bar plots show the percent distribution of green genes with and associated monoallelic or biallelic associated MoI. For figures a), c), d), e) and f), Odds Ratios were calculated by unconditional maximum likelihood estimation (Wald) and confidence intervals (CI) using the normal approximation, with the corresponding adjusted P-values for the Fisher’s exact test. The OR analysis was performed comparing each subset of disease-associated genes versus the overall set of non-disease genes according to OMIM, ORPHANET, DDG2P and GEL-ALL. GEL, Genomics England; PD, Paediatric disorders; ID, Intellectual disability; DDD, Deciphering Developmental Disorders; FA foetal anomalies, CL, cellular lethal, pink; DL, developmental lethal, orange; SV, subviable, yellow; VP, viable with phenotypic abnormalities, light blue; VN, viable with normal phenotype, dark blue. Human diagnostic-grade genes, genes with a high level of evidence for the gene-disease association, as curated by Genomics England and incorporated in its PanelAPP, green genes (see Methods).

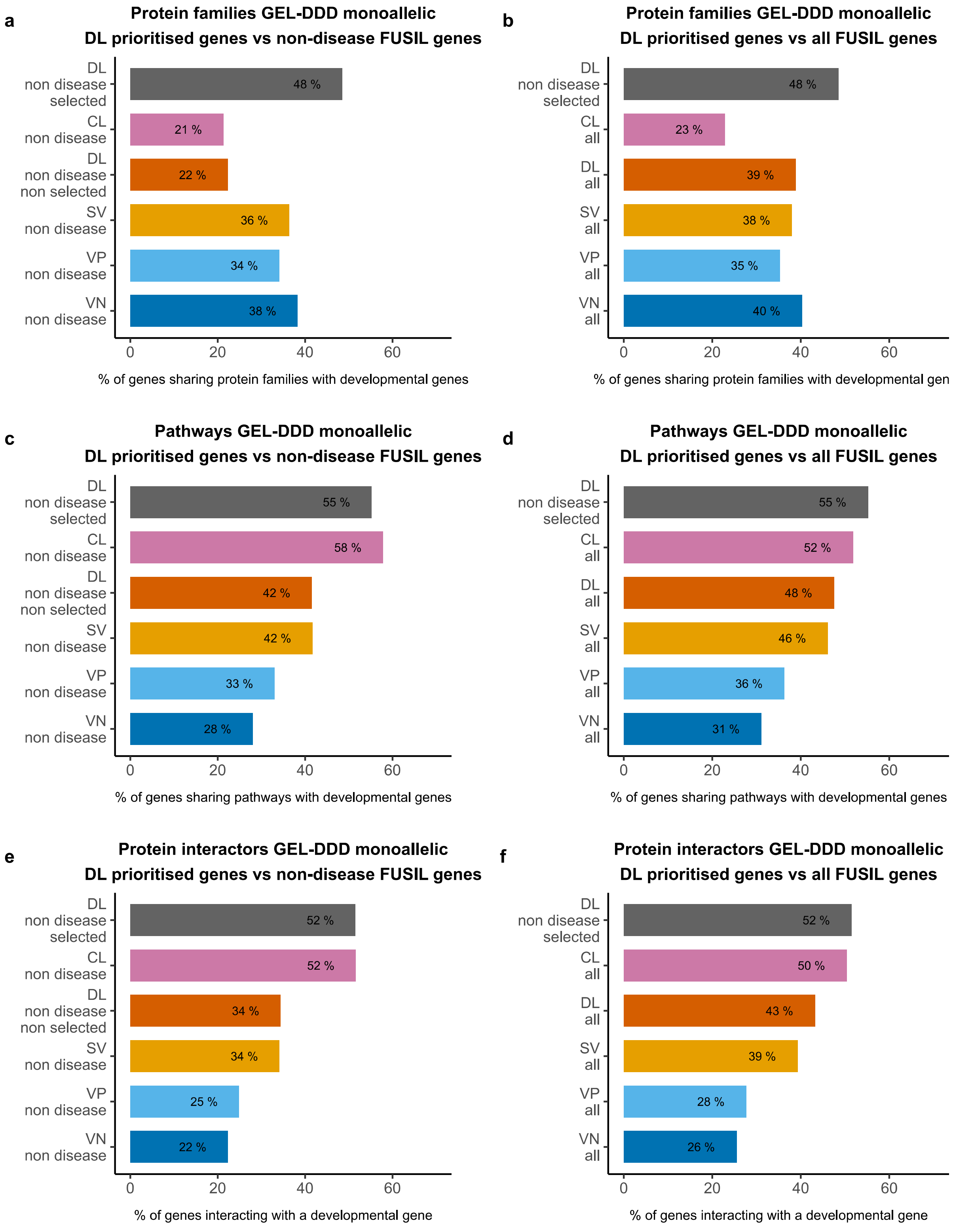

Supplementary Figure 5. Protein family, pathway and interactors analysis of 163 prioritised DL genes.

a) Analysis of PFAM protein families. Bar plots showing the percentage of genes in each category sharing a PFAM[8] protein family with any monoallelic developmental disease gene. Prioritised DL genes are compared with non-disease genes in the different FUSIL categories. **b) Analysis of PFAM protein families**. Bar plots showing the percentage of genes in each category sharing a PFAM protein family with any monoallelic developmental disease gene. Prioritised DL genes are compared with all FUSIL genes. **c) Analysis of Reactome pathways**. Bar plots showing the percentage of genes in each category sharing a Reactome[2] pathway (lowest level) with any monoallelic developmental disease gene. Prioritised DL genes are compared with non-disease genes in the different FUSIL categories. **d) Analysis of Reactome pathways**. Bar plots showing the percentage of genes in each category sharing a Reactome pathway (lowest level) with any monoallelic developmental disease gene. Prioritised DL genes are compared with all genes in the FUSIL bins. **e) Analysis of protein-protein interactors.** Bar plots showing the percentage of genes in each category directly interacting (STRING[9] ppI annotations with a combined score > 0.7) with any monoallelic developmental disease gene. Prioritised DL genes are compared with non-disease genes in the different FUSIL categories. **f) Analysis of protein-protein interactors.** Bar plots showing the percentage of genes in each category directly interacting (STRING[9] ppI annotations with a combined score > 0.7) with any monoallelic developmental disease gene. Prioritised DL genes are compared with all genes in the different FUSIL categories. DL non disease selected, set of 163 prioritised developmental lethal genes, which are a subset of the DL genes not associated to disease, grey; CL non disease, cellular lethal genes not associated to disease (n=258), pink; DL non selected non disease, developmental lethal genes non associated to disease that were not prioritised (n=224) orange; SV non disease, subviable genes not associated to disease (n=264), yellow; VP non disease, viable with phenotypic abnormalities genes not associated to disease (n=1,411), light blue; VN non disease, viable with normal phenotype genes not associated to disease (n=264), dark blue; CL all, cellular lethal (n=413), pink; DL all developmental lethal (n=764), orange; SV all, subviable (n=421), yellow; VP all, viable with phenotypic abnormalities (n=1,867), light blue; VN all, viable with normal phenotype (n=318), dark blue. A set of monoallelic genes from Genomics England DDG2P (GEL-DDD) gene panel was used as reference (n=291).

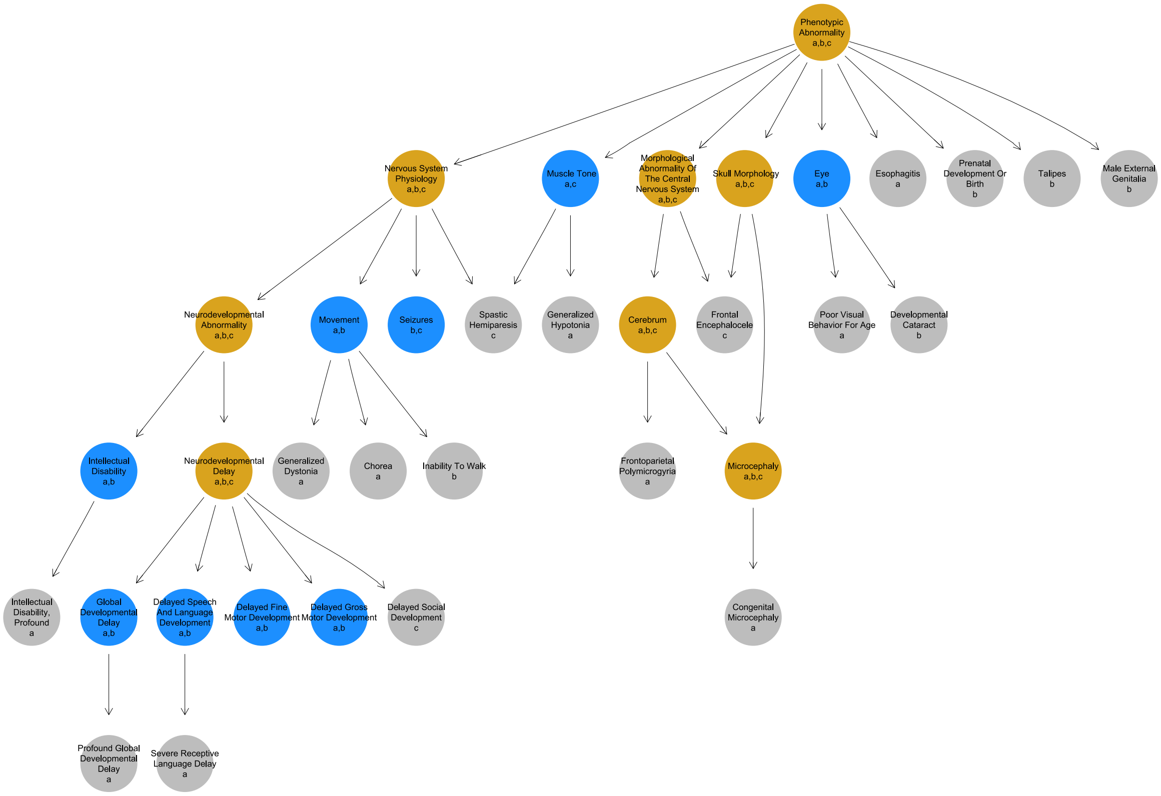

Supplementary Figure 6. HPO phenotypes for *VPS4A* cases.

The set of HPO encoded phenotypes reported for each case listed in Supplementary Table 7 was plotted as a subgraph of the ontology using the R package *ontologyPlot* [10]. Uninformative terms (those annotated to the same objects as all their children) were removed. a: 100KGP patient 1, b: 100KGP patient 2, c: CMG patient. The colour indicates whether the phenotype has been observed in 3 (orange), 2 (blue) or only 1 (grey) patient. For patient c, the original reported phenotypes were replaced by either the synonymous term or the closest term in the HPO (seizures: epilepsy; fontal encephalocele: frontoecephalocele; spastic hemiparesis: right spastic hemiparesis; delayed social development: psychosocial retardation).

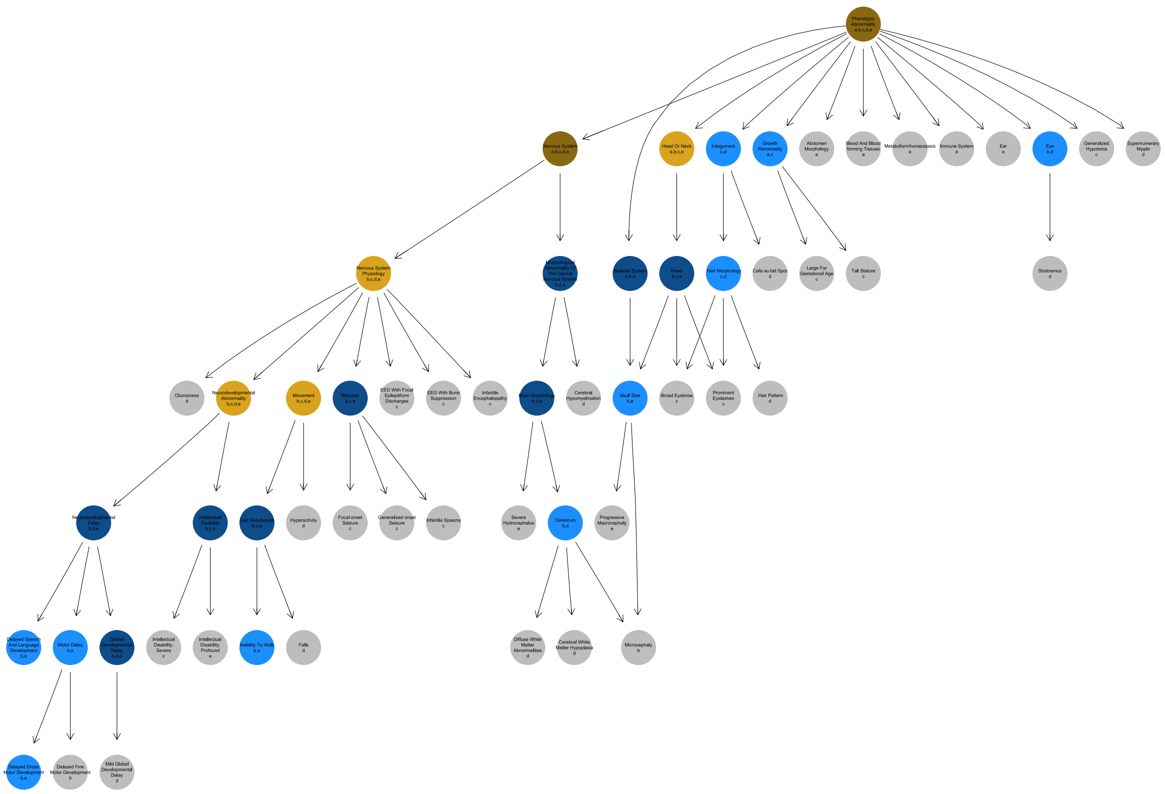

Supplementary Figure 7. HPO phenotypes for *TMEM63B* cases.

The set of HPO encoded phenotypes reported for each case listed in Supplementary Table 8 was plotted as a subgraph of the ontology using the R package *ontologyPlot*[10]. Uninformative terms (those annotated to the same objects as all their children) were removed. a: DDD patient 1, b: 100KGP patient 1, c: 100KGP patient 2, d: 100KGP patient 3, e: 100KGP patient 4. The colour indicates whether the phenotype has been observed in 5 (dark orange), 4 (orange), 3 (dark blue), 2 (blue) or only 1 (grey) patient.

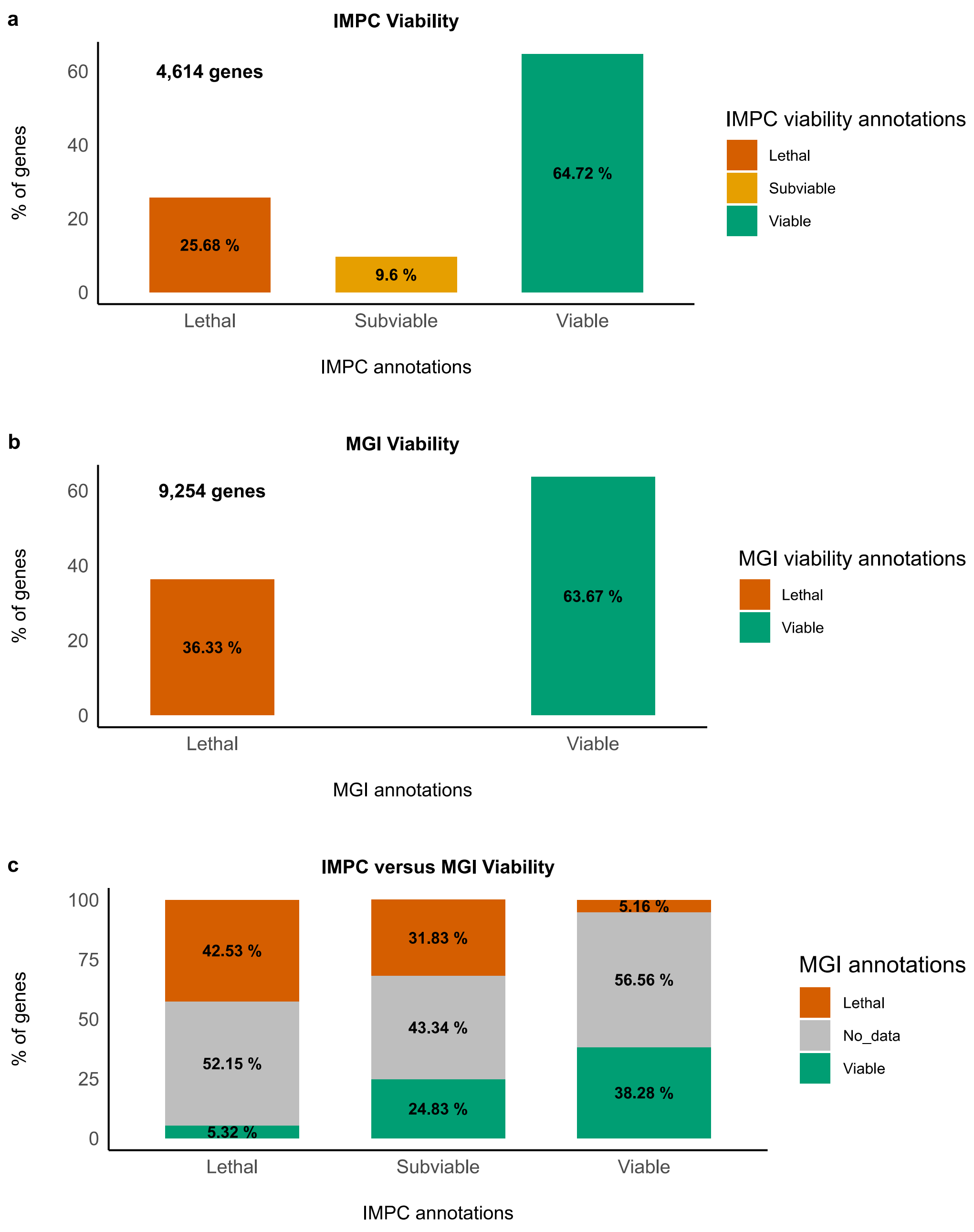

Supplementary Figure 8. Evidence for mouse viability for the genes considered in this study and genes annotated in MGI.

a) IMPC Viability. Bar plots showing the percent distribution of primary viability assessment outcomes as obtained from the IMPC (Table 1, Methods). **b) MGI Viability.** Bar plots showing the percent distribution of viability annotations as obtained from Mouse Genome Informatics (MGI)[11]. Gene to phenotype annotations (excluding conditional annotations) from MGI were used to identify the set of genes with embryo lethality phenotypes (50 Mammalian Phenotype

Ontology terms as described in Dickinson, et al. [12]; viability outcomes inferred from MGI annotations do not include the IMPC subviable category**. c) Correspondence between IMPC and MGI annotations.** For each IMPC viability category, the bar plots represent the percentage distribution of the viability assessment according to MGI annotations. For 2,115 mouse genes with both IMPC and non-IMPC phenotypic annotations available to infer viability, we found discrepancies for a set of 63 genes that were found to be lethal according to the IMPC but had no previous records of lethality in MGI as well as for 154 genes viable as reported by the IMPC and with some type of lethality annotations reported in MGI (10% overall discrepancy). IMPC, International Mouse Phenotyping Consortium; MGI, Mouse Genome Informatics.

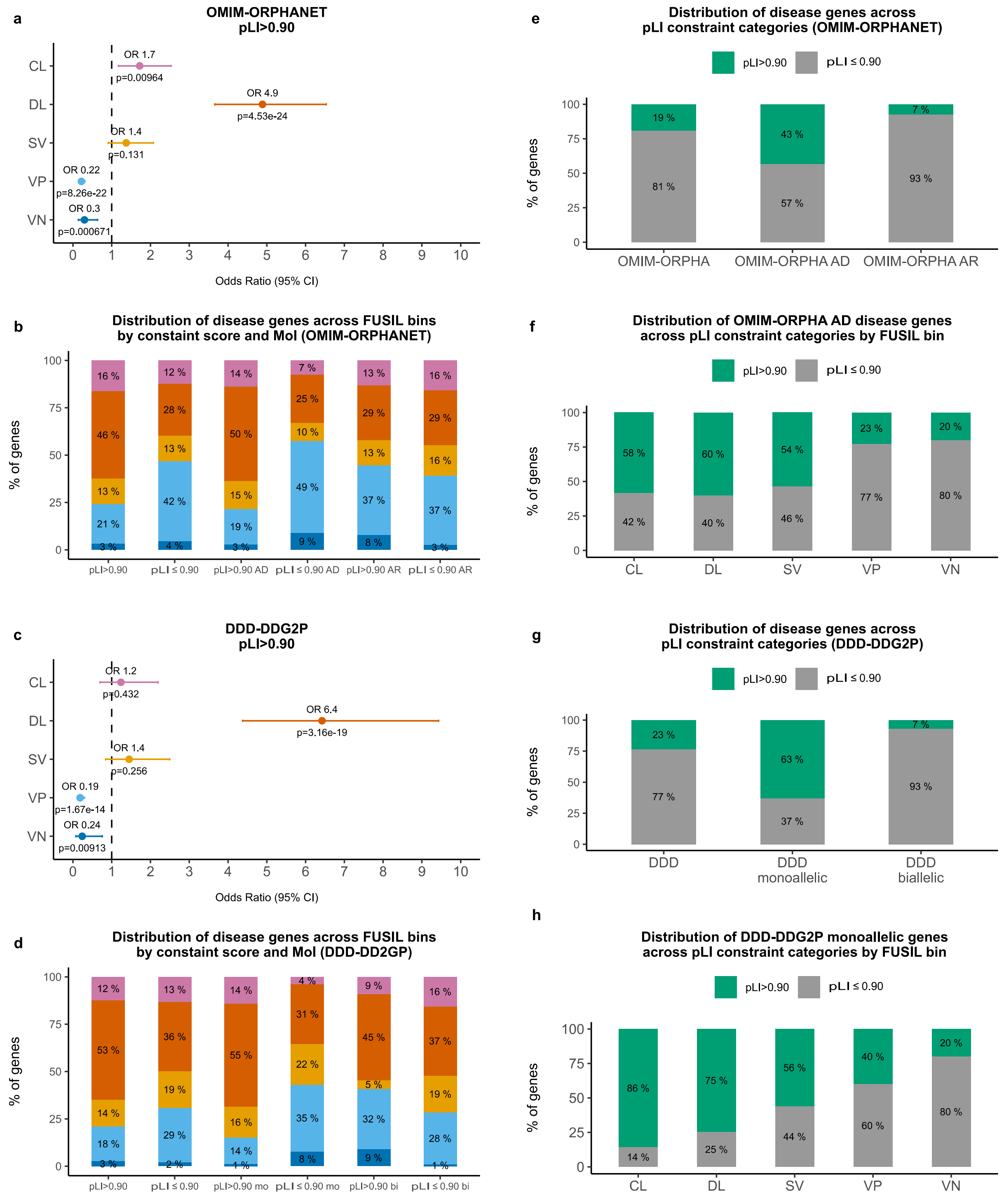

Supplementary Figure 9. Integration of FUSIL categories with constraint scores.

a) Enrichment analysis of highly constrained Mendelian disease genes. Combined OMIM-ORPHANET data was used to compute the number of disease genes in each FUSIL bin with a gnomAD pLI score>0.90. The genes meeting these criteria were compared to non-disease

genes. **b) Distribution of Mendelian disease genes across FUSIL bins by constraint score and MoI**. Percent distribution of OMIM-ORPHANET Mendelian disease genes according to constraint score and mode of inheritance**. c) Enrichment analysis of highly constrained developmental disorder genes**. DDD-DDG2P set of genes was used to compute the number of developmental disorder genes in each FUSIL bin with a gnomAD pLI score>0.90. The genes meeting these criteria were compared to non-disease genes. **d) Distribution of developmental disorder genes across FUSIL bins by constraint score and MoI.** Percent distribution of DDD-DDG2P developmental disease genes according to constraint score and mode of inheritance. **e) Distribution of Mendelian disease genes across pLI constraint categories by MoI**. Percent distribution of OMIM-ORPHANET Mendelian disease genes across two pLI constraint categories (highly constraint genes, pLI >0.90) according to mode of inheritance. **f) Distribution of Mendelian disease genes across pLI constraint categories by FUSIL bin**. Percent distribution of OMIM-ORPHANET AD Mendelian disease genes across two pLI constraint categories (highly constraint genes, pLI >0.90) by FUSIL category. **g) Distribution of developmental disorder genes across pLI constraint categories by MoI**. Percent distribution of DDD-DD2GP genes across two pLI constraint categories (highly constraint genes, pLI >0.90) according to allelic requirement. **h) Distribution of developmental disorder genes across pLI constraint categories by FUSIL bin**. Percent distribution of DDD-DD2GP monoallelic genes across two pLI constraint categories (highly constraint genes, pLI >0.90) by FUSIL category.

For figures a) and c) Odds Ratios were calculated by unconditional maximum likelihood estimation (Wald) and confidence intervals (CI) using the normal approximation, with the corresponding adjusted P-values for the Fisher’s exact test.

For figures e) and g) percentages are computed based on the subset of genes with FUSIL information. CL, cellular lethal, pink; DL, developmental lethal, orange; SV, subviable, yellow; VP, viable with phenotypic abnormalities, light blue; VN, viable with normal phenotype, dark blue; DDD-DD2GP, Deciphering Developmental Disorders database of genes likely causative of developmental disorders; MoI, mode of inheritance; pLI, probability of being loss of function intolerant; AD, autosomal dominant; AR, autosomal recessive; mo, monoallelic; bi, biallelic.

Supplementary Table 1. **FUSIL categories.** Classification of genes based on KO mice viability assessment and phenotypes as obtained by the IMPC and human cell essentiality scores (Avana) as obtained from the Project Achilles (see methods for full details). In bold, data shown in Table 1.

Mouse viability phenotype	Human cell essentiality score	FUSIL category	Class	Number of genes
Lethal	≤ -0.45	**Cellular lethal (CL)**	Lethal in mouse and essential in human cell lines	**413**
	> -0.45	**Developmental lethal (DL)**	Lethal in mouse but non-essential in human cell lines	**764**
Subviable	≤ -0.45	Subviable outlier (SV.outlier)	Subviable in mouse and essential in human cell lines	16
	> -0.45	**Subviable (SV)**	Subviable in mouse and non-essential in human cell lines	**421**
Viable	> -0.45	**Viable with phenotype (VP)**	Viable and non-essential in human cells (at least one significant phenotype it in the adult homozygous null mice)	**1,867**
	> -0.45	**Viable with no phenotype (VN)**	Viable and non-essential in human cells (no significant phenotype hits in the adult homozygous null mice when % procedures done ≥ 50%)	**318**
	> -0.45	Viable insufficient data on procedures (V. insuffProcedures)	Viable and non-essential in human cells (no significant phenotype hits in the adult homozygous null mice when % procedures done < 50% / difficult to ascertain)	627
	≤ -0.45	Viable outlier (V.outlier)	Viable in the mouse & essential in human cells	20

Supplementary Table 2. **Human cell essentiality assessment**. Comparison between the set of essential and non-essential genes based on mean Avana CRISPR-Cas9 screens performed on over 400 cell lines and 11 cell lines from 3 different studies (see Supplementary Figure 1). For any given gene, a mean Avana score ≤ -0.45 resulted in considering the gene essential.

Mean Avana -0.45 threshold	11 cell lines	Number of overlapping genes	% Overlap	% total
Essential	Essential	1,339	79.85 %	96.11 %
Essential	Non-essential	338	20.15 %	
Non-essential	Essential	312	2.07 %	
Non-essential	Non-essential	14,751	97.93 %	

Supplementary Table 3. **Embryo windows of lethality**. Embryonic viability assessment outcomes indicate the embryonic stage at which the homozygous LoF mice manifested lethality and their overlap with human cell essentiality categories. E, embryonic day.

<table>
<tr><th rowspan="2">Mouse embryonic group</th><th rowspan="2">Windows of embryo lethality</th><th rowspan="2">Total number of genes</th><th rowspan="2">%</th><th colspan="2">Genes with human cell essentiality information (%)</th></tr>
<tr><th>Essential</th><th>Non-essential</th></tr>
<tr><td>Early gestation</td><td>prior to E9.5</td><td>197</td><td>49.25%</td><td>125
(64.76%)</td><td>68
(35.23%)</td></tr>
<tr><td rowspan="2">Mid gestation</td><td>E9.5-E12.5</td><td>45</td><td rowspan="2">12.50%</td><td rowspan="2">5
(10.20%)</td><td rowspan="2">44
(89.80%)</td></tr>
<tr><td>E12.5-E14.5/E15.5</td><td>5</td></tr>
<tr><td rowspan="3">Late gestation</td><td>E14.5/E15.5-E18.5</td><td>3</td><td rowspan="3">38.25%</td><td rowspan="3">7
(4.70%)</td><td rowspan="3">142
(95.30%)</td></tr>
<tr><td>after E14.5/E15.5</td><td>75</td></tr>
<tr><td>after E18.5</td><td>75</td></tr>
</table>

Supplementary Table 4. Gene features. Adjusted P-values (Wilcoxon test, two-sided, Benjamini and Hochberg correction) for all pairwise comparisons (boxplots in Fig. 2).

FUSIL bin 1	FUSIL bin 2	Recomb Rate	TPM Brain Cortex	TPM Cells Transform Fibroblasts	TPM Ovary	TPM Testis	Degree	Topological Coefficient	Probability of mutation	Transcript length	GIMS Selection Score
CL	DL	5.7E-16	1.8E-06	3.3E-15	4.9E-09	5.5E-19	1.4E-16	3.1E-03	4.0E-01	3.3E-02	7.8E-01
CL	SV	5.3E-15	2.1E-10	8.0E-21	3.3E-14	1.4E-20	2.0E-17	6.7E-06	6.0E-02	1.2E-04	7.8E-01
CL	VP	1.0E-33	6.4E-30	3.4E-77	4.1E-51	2.5E-57	5.2E-46	2.3E-12	1.7E-01	3.4E-01	5.6E-18
CL	VN	8.0E-21	8.0E-22	1.0E-51	9.0E-38	9.9E-39	2.2E-24	3.9E-10	3.5E-02	7.7E-02	5.0E-12
DL	SV	4.8E-01	2.0E-02	1.0E-03	2.6E-03	1.7E-02	1.3E-02	4.0E-02	1.3E-01	3.2E-02	7.8E-01
DL	VP	2.3E-04	3.4E-16	6.9E-43	1.1E-31	1.4E-20	5.0E-16	6.7E-06	4.8E-03	4.4E-05	1.2E-30
DL	VN	1.0E-02	1.2E-11	4.9E-28	1.7E-22	4.5E-15	3.4E-08	4.9E-05	1.4E-03	1.1E-04	1.0E-15
SV	VP	4.0E-02	2.2E-05	1.1E-12	2.9E-09	4.3E-06	2.6E-04	2.3E-01	3.9E-05	2.8E-08	1.2E-20
SV	VN	8.8E-02	7.5E-06	5.3E-13	3.2E-10	7.9E-07	1.7E-03	6.4E-02	2.8E-05	2.0E-07	2.2E-13
VP	VN	9.1E-01	1.0E-01	6.2E-03	1.3E-02	5.1E-02	4.9E-01	2.4E-01	1.3E-01	1.9E-01	5.7E-01

Supplementary Table 5. **Constraint scores**. Adjusted P-values (Wilcoxon test, two-sided, Benjamini and Hochberg correction) for all pairwise comparisons (boxplots in Supplementary Figure 2).

FUSIL bin 1	FUSIL bin 2	pLI	o/e LoF	o/e LoF upper bound (LOEUF)	o/e mis	o/e syn	shet	RVIS	HI
CL	DL	1.1E-01	4.1E-01	5.0E-01	5.6E-01	9.6E-01	8.9E-01	1.4E-01	3.7E-01
CL	SV	1.6E-01	6.8E-01	3.6E-01	5.6E-01	5.2E-01	5.8E-01	7.9E-01	1.2E-01
CL	VP	9.7E-12	3.4E-21	4.7E-23	3.3E-14	2.1E-01	8.1E-19	1.8E-20	9.2E-42
CL	VN	2.2E-08	1.2E-16	2.3E-21	2.0E-11	5.2E-01	1.8E-15	6.7E-18	2.8E-28
DL	SV	9.7E-01	6.8E-01	7.3E-01	9.1E-01	5.2E-01	5.6E-01	9.8E-02	3.7E-01
DL	VP	5.3E-28	1.1E-38	2.0E-38	5.8E-20	1.3E-01	9.4E-26	1.8E-20	1.3E-52
DL	VN	2.7E-15	4.6E-23	2.7E-27	4.0E-13	5.2E-01	2.1E-17	1.6E-15	1.2E-28
SV	VP	1.6E-17	1.8E-22	3.0E-26	3.3E-13	5.2E-01	7.7E-21	9.6E-20	3.6E-29
SV	VN	4.9E-12	2.0E-17	3.8E-23	7.0E-11	9.6E-01	2.1E-17	1.1E-16	6.3E-21
VP	VN	7.2E-01	1.3E-01	3.8E-03	2.0E-01	5.2E-01	5.3E-02	7.5E-02	1.2E-01

Supplementary Table 6. **Clinical features for AD disease genes across FUSIL bins.**

Distribution of autosomal dominant disease genes across FUSIL bins based on the number of physiological systems affected and the age of onset (only those genes with information for all three features were considered for this analysis). MoI, mode of inheritance; N, number of genes; number of physiological systems affected: high (≥13), intermediate (6-13), low (≤6). Age of onset: early (antenatal, neonatal), intermediate (infancy, childhood), late (other).

FUSIL bin	MoI	Number of physiological systems affected	Age of onset	N	N FUSIL	N FUSIL and MoI	% (FUSIL)	% (FUSIL and MoI)
CL	AD	high	early	5	110	22	4.55	22.73
CL	AD	high	intermediate	2	110	22	1.82	9.09
CL	AD	high	late	0	110	22	0	0
CL	AD	intermediate	early	2	110	22	1.82	9.09
CL	AD	intermediate	intermediate	5	110	22	4.55	22.73
CL	AD	intermediate	late	3	110	22	2.73	13.64
CL	AD	low	early	1	110	22	0.91	4.55
CL	AD	low	intermediate	3	110	22	2.73	13.64
CL	AD	low	late	1	110	22	0.91	4.55
DL	AD	high	early	24	264	82	9.09	29.27
DL	AD	high	intermediate	6	264	82	2.27	7.32
DL	AD	high	late	2	264	82	0.76	2.44
DL	AD	intermediate	early	16	264	82	6.06	19.51
DL	AD	intermediate	intermediate	6	264	82	2.27	7.32
DL	AD	intermediate	late	6	264	82	2.27	7.32
DL	AD	low	early	9	264	82	3.41	10.98
DL	AD	low	intermediate	9	264	82	3.41	10.98
DL	AD	low	late	4	264	82	1.52	4.88
SV	AD	high	early	8	113	22	7.08	36.36
SV	AD	high	intermediate	1	113	22	0.88	4.55
SV	AD	high	late	0	113	22	0	0
SV	AD	intermediate	early	6	113	22	5.31	27.27
SV	AD	intermediate	intermediate	1	113	22	0.88	4.55
SV	AD	intermediate	late	1	113	22	0.88	4.55
SV	AD	low	early	2	113	22	1.77	9.09
SV	AD	low	intermediate	1	113	22	0.88	4.55
SV	AD	low	late	2	113	22	1.77	9.09
VP	AD	high	early	8	288	70	2.78	11.43
VP	AD	high	intermediate	3	288	70	1.04	4.29
VP	AD	high	late	4	288	70	1.39	5.71
VP	AD	intermediate	early	12	288	70	4.17	17.14
VP	AD	intermediate	intermediate	5	288	70	1.74	7.14
VP	AD	intermediate	late	9	288	70	3.12	12.86
VP	AD	low	early	6	288	70	2.08	8.57
VP	AD	low	intermediate	8	288	70	2.78	11.43
VP	AD	low	late	15	288	70	5.21	21.43
VN	AD	high	early	0	28	14	0	0
VN	AD	high	intermediate	0	28	14	0	0
VN	AD	high	late	0	28	14	0	0
VN	AD	intermediate	early	2	28	14	7.14	14.29

VN	AD	intermediate	intermediate	3	28	14	10.71	21.43
VN	AD	intermediate	late	1	28	14	3.57	7.14
VN	AD	low	early	1	28	14	3.57	7.14
VN	AD	low	intermediate	3	28	14	10.71	21.43
VN	AD	low	late	4	28	14	14.29	28.57

Supplementary Table 7. Clinical description of patients with variants in *VPS4A.*

Phenotypes reported for each patient, shared phenotypes in bold.

	100KGP patient 1	100KGP patient 2	CMG patient
de novo variant	16:69320768:A:T (GRCh38)	16:69319539:G:A (GRCh38)	Variant data unavailable
Behavioural phenotypes	• **Intellectual disability, profound**<br>• **Profound global developmental delay**<br>• **Severe receptive language delay**	• **Intellectual disability**<br>• **Global developmental delay**<br>• **Delayed speech and language development**<br>• Abnormality of prenatal development or birth	• **Psychosocial retardation**
Movement / Muscle phenotypes	• **Delayed fine motor development**<br>• **Delayed gross motor development**<br>• Generalized hypotonia<br>• Generalized dystonia<br>• Chorea	• **Delayed fine motor development**<br>• **Delayed gross motor development**<br>• Inability to walk	• Right spastic hemiparesis
Seizure phenotypes		• **Seizures**	• **Epilepsy**
Other brain phenotypes	• **Congenital microcephaly**<br>• Frontoparietal polymicrogyria	• **Microcephaly**<br>• Morphological abnormality of the central nervous system	• **Microcephaly**<br>• Frontoencephalocele
Other phenotypes	• **Poor visual behavior for age**<br>• Esophagitis	• **Abnormality of the eye**<br>• **Developmental cataract**<br>• Talipes<br>• Abnormality of male external genitalia	

Supplementary Table 8. Clinical description of patients with variants in *TMEM63B*.

Phenotypes reported for each patient, shared phenotypes in bold.

	DDD patient 1	100KGP patient 1	100KGP patient 2	100KGP patient 3	100KGP patient 4
de novo variant	6:44134714:G:A (GRCh38)			6:44151868:G:A (GRCh38)	6:44148860:TCC:: (GRCh38)
Behavioural phenotypes	• Abnormality of the nervous system	• **Intellectual disability**<br>• **Global developmental delay**<br>• **Delayed speech and language development**	• **Intellectual disability, severe**	• **Mild global developmental delay**<br>• Hyperactivity	• **Intellectual disability, profound**<br>• **Global developmental delay**<br>• **Delayed speech and language development**
Movement phenotypes	• Abnormality of the nervous system	• **Delayed gross motor development**<br>• **Inability to walk**<br>• Delayed fine motor development	• Generalized hypotonia<br>• Abnormality of movement	• Clumsiness<br>• Falls	• **Delayed gross motor development**<br>• **Inability to walk**
Seizure phenotypes	• Abnormality of the nervous system	• **Seizures**	• **Focal-onset seizure**<br>• **Generalized-onset seizure**<br>• **Infantile spasms**<br>• **EEG with focal epileptiform discharges**<br>• **EEG with generalized epileptiform discharges**<br>• **EEG with burst suppression**		• **Seizures**
Other brain phenotypes	• Abnormality of the nervous system<br>• Abnormality of head or neck	• Microcephaly<br>• Morphological abnormality of the central nervous system	• Infantile encephalopathy	• Cerebral hypomyelination<br>• Cerebral white matter hypoplasia<br>• Diffuse white matter abnormalities	• Progressive macrocephaly<br>• Severe hydrocephalus
Other phenotypes	• **Growth abnormality**<br>• Abnormality of the skeletal system<br>• Abnormality of abdomen morphology<br>• Abnormality of blood and blood-forming tissues<br>• Abnormality of metabolism/homeostasis<br>• Abnormality of the immune system<br>• Abnormality of the ear	• **Abnormality of the eye**	• Large for gestational age<br>• **Tall stature**<br>• Prominent eyelashes<br>• Broad eyebrow	• **Strabismus**<br>• Supernumerary nipple<br>• Cafe-au-lait spot<br>• Abnormal hair pattern	

Supplementary Table 9. **IMPC and MGI viability assessment.** IMPC viability outcomes compared to MGI reported phenotypes.

IMPC Viability (primary viability assessment)	MGI Viability (reported phenotypes)	Number of genes	% of discrepancy with respect to IMPC Viability category
Lethal	Lethal	504	
Lethal	Viable	63	11.11%
Subviable	Lethal	141	-
Subviable	Viable	110	-
Viable	Lethal	154	11.87%
Viable	Viable	1,143	

Supplementary References

1. Munoz-Fuentes, V. *et al.* The International Mouse Phenotyping Consortium (IMPC): a functional catalogue of the mammalian genome that informs conservation. *Conservation Genetics* **19**, 995-1005 (2018).
2. Fabregat, A. *et al.* The Reactome Pathway Knowledgebase. *Nucleic Acids Res* **46**, D649-D655 (2018).
3. Karczewski, K.J. *et al.* Variation across 141,456 human exomes and genomes reveals the spectrum of loss-of-function intolerance across human protein-coding genes. *Preprint at* https://www.biorxiv.org/content/10.1101/531210v3 (2019).
4. Cummings, B.B. *et al.* Transcript expression-aware annotation improves rare variant discovery and interpretation. *Preprint at* https://www.biorxiv.org/content/10.1101/554444v1 (2019).
5. Cassa, C.A. *et al.* Estimating the selective effects of heterozygous protein-truncating variants from human exome data. *Nat Genet* **49**, 806-810 (2017).
6. Petrovski, S., Wang, Q., Heinzen, E.L., Allen, A.S. & Goldstein, D.B. Genic intolerance to functional variation and the interpretation of personal genomes. *PLoS Genet* **9**, e1003709 (2013).
7. Huang, N., Lee, I., Marcotte, E.M. & Hurles, M.E. Characterising and predicting haploinsufficiency in the human genome. *PLoS Genet* **6**, e1001154 (2010).
8. El-Gebali, S. *et al.* The Pfam protein families database in 2019. *Nucleic Acids Res* **47**, D427-D432 (2019).
9. Szklarczyk, D. *et al.* STRING v10: protein-protein interaction networks, integrated over the tree of life. *Nucleic Acids Research* **43**, D447-D452 (2015).
10. Greene, D. ontologyPlot: Functions for Visualising Sets of Ontological Terms. R package version 1.4. (2016).
11. Smith, C.L. *et al.* Mouse Genome Database (MGD)-2018: knowledgebase for the laboratory mouse. *Nucleic Acids Research* **46**, D836-D842 (2018).
12. Dickinson, M.E. *et al.* High-throughput discovery of novel developmental phenotypes. *Nature* **537**, 508-514 (2016).
