# Supplementary material for: Human and mouse essentiality screens as a resource for disease gene discovery
Source: Nat Commun. 2020 Jan 31;11:655. doi: 10.1038/s41467-020-14284-2 (PMC6994715; doi:10.1038/s41467-020-14284-2)
Supplement: Supplementary file 3 — Reporting Summary [file 41467_2020_14284_MOESM3_ESM.pdf]

nature research

Corresponding author(s): Damian Smedley

Last updated by author(s): Dec 9, 2019

Reporting Summary

Nature Research wishes to improve the reproducibility of the work that we publish. This form provides structure for consistency and transparency in reporting. For further information on Nature Research policies, see Authors & Referees and the Editorial Policy Checklist .

Statistics

For all statistical analyses, confirm that the following items are present in the figure legend, table legend, main text, or Methods section.

n/a	Confirmed	
☐	☒	The exact sample size (*n*) for each experimental group/condition, given as a discrete number and unit of measurement
☐	☒	A statement on whether measurements were taken from distinct samples or whether the same sample was measured repeatedly
☐	☒	The statistical test(s) used AND whether they are one- or two-sided<br>*Only common tests should be described solely by name; describe more complex techniques in the Methods section.*
☐	☒	A description of all covariates tested
☐	☒	A description of any assumptions or corrections, such as tests of normality and adjustment for multiple comparisons
☐	☒	A full description of the statistical parameters including central tendency (e.g. means) or other basic estimates (e.g. regression coefficient) AND variation (e.g. standard deviation) or associated estimates of uncertainty (e.g. confidence intervals)
☐	☒	For null hypothesis testing, the test statistic (e.g. *F*, *t*, *r*) with confidence intervals, effect sizes, degrees of freedom and *P* value noted<br>*Give P values as exact values whenever suitable.*
☒	☐	For Bayesian analysis, information on the choice of priors and Markov chain Monte Carlo settings
☒	☐	For hierarchical and complex designs, identification of the appropriate level for tests and full reporting of outcomes
☐	☒	Estimates of effect sizes (e.g. Cohen's *d*, Pearson's *r*), indicating how they were calculated

Our web collection on statistics for biologists contains articles on many of the points above.

Software and code

Policy information about availability of computer code

Data collection	Mouse Phenotyping Informatics Infrastructure (MPI2) pipeline (https://github.com/mpi2) , PhenStat (Bioconductor R package)
Data analysis	R software (R-3.5.1) was used for data analysis and visualisation

For manuscripts utilizing custom algorithms or software that are central to the research but not yet described in published literature, software must be made available to editors/reviewers. We strongly encourage code deposition in a community repository (e.g. GitHub). See the Nature Research guidelines for submitting code & software for further information.

Data

Policy information about availability of data

All manuscripts must include a data availability statement. This statement should provide the following information, where applicable:

- Accession codes, unique identifiers, or web links for publicly available datasets
- A list of figures that have associated raw data
- A description of any restrictions on data availability

Data presented in this study are available in public repositories (ftp://ftp.ebi.ac.uk/pub/databases/impc/ and https://www.ebi.ac.uk/biostudies/studies/S-BSST293)

Field-specific reporting

Please select the one below that is the best fit for your research. If you are not sure, read the appropriate sections before making your selection.

☒ Life sciences ☐ Behavioural & social sciences ☐ Ecological, evolutionary & environmental sciences

For a reference copy of the document with all sections, see nature.com/documents/nr-reporting-summary-flat.pdf

Life sciences study design

All studies must disclose on these points even when the disclosure is negative.

Sample size	All the IMPC viability data according to DR9.1 was used for the analysis. The sample size of the different categories ranged from 318 to 1,867.
Data exclusions	Data exclusions from the original DR9.1 viability report are extensively explained in the Methods section (conflicting calls / low confidence orthologs...)
Replication	The IMPC uses standardized mouse genotypes along with standardized viability and phenotyping assay protocols across centers to maximize replicability
Randomization	Viability assessment (lethal, subviable, viable) is based on the number of homozygous (null and wild type) and heterozygous pups
Blinding	Mouse pups are genotyped before weaning

Reporting for specific materials, systems and methods

We require information from authors about some types of materials, experimental systems and methods used in many studies. Here, indicate whether each material, system or method listed is relevant to your study. If you are not sure if a list item applies to your research, read the appropriate section before selecting a response.

Materials & experimental systems

n/a	Involved in the study
☒	☐ Antibodies
☒	☐ Eukaryotic cell lines
☒	☐ Palaeontology
☐	☒ Animals and other organisms
☐	☒ Human research participants
☒	☐ Clinical data

Methods

n/a	Involved in the study
☒	☐ ChIP-seq
☒	☐ Flow cytometry
☒	☐ MRI-based neuroimaging

Animals and other organisms

Policy information about studies involving animals; ARRIVE guidelines recommended for reporting animal research

Laboratory animals	Male and female mouse knockouts on an isogenic genetic background (C57BL/6N)
Wild animals	The study did not involve wild animals
Field-collected samples	The study did not involve samples collected from the field
Ethics oversight	Mouse production, breeding and phenotyping at each centre was done in compliance with each centre's ethical animal care and use guidelines in addition to the applicable licensing and accrediting bodies, in accordance with the national legislation under which each centre operates (Animal Welfare and Ethical Review Body (AWERB), UC Davis Institutional Animal Care and Use Committee (IACUC), Animal Care Committee (ACC) of the Toronto Centre for Phenogenomics, IACUC of MARC, The RIKEN Tsukuba Animal Experiments Committee, Com'Eth. agreement nb: 17, Regierung von Oberbayern, The Jackson Laboratory Institutional Animal Care and Use Committee (IACUC), Institutional Animal Care and Usage Committee) .

Note that full information on the approval of the study protocol must also be provided in the manuscript.

Human research participants

Policy information about studies involving human research participants

Population characteristics	100KGP participants: patients affected by a rare disease (or cancer) from the general UK population
Recruitment	100KGP participants: National Health Service (NHS) patients and their family members who entered the 100KGP project
Ethics oversight	The 100KGP research and clinical project model and its informed consent process has been approved by the National Research Ethics Service Research Ethics Committee for East of England – Cambridge South Research Ethics Committee.

Note that full information on the approval of the study protocol must also be provided in the manuscript.
