# Supplementary material for: Human and mouse essentiality screens as a resource for disease gene discovery
Source: Nat Commun. 2020 Jan 31;11:655. doi: 10.1038/s41467-020-14284-2 (PMC6994715; doi:10.1038/s41467-020-14284-2)
Supplement: Supplementary file 4 — Description of Additional Supplementary Files [file 41467_2020_14284_MOESM4_ESM.pdf]

Title: ***Supplementary Data 1. Gene Ontology (GO) Biological Process (BP) enrichment***.

Description: *Set of Gene Ontology Biological Processes with significant results after correcting for multiple testing for the CL and DL category of genes (Fig. 1b). Genes in the SV and viable categories (VP, VN) were not significantly enriched in any biological process despite reasonable sample sizes, probably reflecting diverse roles for these genes.*

Title: ***Supplementary Data 2. Reactome pathways enrichment***.

Description: *Set of Reactome pathways with significant results after correcting for multiple testing. Visualization of enriched pathways for the CL and DL categories is shown in Supplementary Fig. 2.*

Title: ***Supplementary Data 3. 163 DL genes that are highly intolerant to loss-of-function variation and not currently associated with human disease and their relationship with known monoallelic developmental genes.***

Description: *Those genes sharing a pathway, a protein family of directly interacting with a known monoallelic developmental disease gene (GEL-DDG2P panel, green genes) are highlighted in bold and the corresponding known developmental genes are annotated.*

Title: ***Supplementary Data 4. 163 DL genes that are highly intolerant to loss-of-function variation and not currently associated with human disease with their corresponding constraint scores and variant annotations***.

Description: *Set of prioritised genes from the list of 764 DL genes, with the corresponding intolerance to variation scores and presence of candidate variants as identified by any of the 3 sequencing programs selected in the current study: Y, candidate variant identified / candidate variant absent from gnomAD; N, candidate variant present in gnomAD; DN, gene with predicted functional de novo variant(s) present in denovo-db database from other sources than DDD; DN (DDD) gene with predicted functional de novo variant(s) present in denovo-db database from the DDD study; DN (DDD +), gene with predicted functional de novo variant(s) present in denovo-db database from DDD and other studies.*
